# Life History Plasticity and Gregarious Cocooning Behavior of the Wild Silkmoth *Cricula trifenestrata* Helfer (Lepidoptera: Saturniidae) on a Novel Host Plant, Cinnamon, in Thailand

**DOI:** 10.3390/insects16090914

**Published:** 2025-09-01

**Authors:** Kanitsara Magnussen, Motoyuki Sumida, Suwat Promma, Anongrit Kangrang, Fritz Vollrath, Thanupong Thunchailertthakul, Chirapha Butiman

**Affiliations:** 1 Department of Biology, Faculty of Science, Mahasarakham University, Kantharawichai District, Maha Sarakham 44150, Thailand; 2 The Research Unit of Center of Excellence for Mulberry and Silk, Center of Excellence for Silk Innovation, Mahasarakham University, Kantharawichai District, Maha Sarakham 44150, Thailand; suwat.p@msu.ac.th (S.P.); chirapha_b@msu.ac.th (C.B.); 3 Center of Excellence for Silk Innovation, Mahasarakham University, Kantharawichai District, Maha Sarakham 44150, Thailand; mtysumida@gmail.com; 4 Department of Environmental Engineering, Faculty of Engineering, Mahasarakham University, Kantharawichai District, Maha Sarakham 44150, Thailand; anongrit.k@msu.ac.th; 5 Department of Biology, University of Oxford, Oxford OX1 3PS, UK; fritz.vollrath@biology.ox.ac.uk; 6 Thip Patthana Mai Thai Ltd., 136/9 Moo 14 Chaengsanit Rd., Khaen Nuea, Ban Phai, Khon Kaen 44110, Thailand

**Keywords:** behavior, cinnamon, cocoon traits, Lepidoptera, life history, Saturniidae, sericulture, wild silkmoth

## Abstract

*Cricula trifenestrata* Helfer was recently documented on cinnamon (*Cinnamomum* spp.), a novel host plant in Thailand. We compared life history and behavior under natural field, semi-natural caged, and laboratory conditions on cinnamon, plus opportunistic collection from hog plum (*Spondias pinnata*). Laboratory rearing significantly extended development (62.30 ± 3.68 days) versus field conditions (56.30 ± 1.83 days, *p* < 0.001) through delayed egg and pupal stages, indicating life history plasticity. We discovered density-dependent oviposition plasticity (linear arrangements in field: 155.6 ± 84.9 eggs/batch; clustered in laboratory) and novel gregarious cocooning behavior, where 85.1% of individuals (*n* = 47 sites) aggregated to form communal clusters with unique reticulated architecture. Female cocoon shell ratio (CSR) was significantly higher in laboratory (5.02 ± 0.72%) and hog plum cohorts (5.30 ± 0.30%) than field conditions (3.92 ± 0.51%, *p* = 0.002). Opportunistic rearing yielded >2 kg fresh cocoons, with clusters reaching 0.220 kg. These findings establish biological baselines for *C. trifenestrata* Helfer on cinnamon, reveal sophisticated social behaviors expanding lepidopteran sociality concepts, and demonstrate commercial potential for sustainable sericulture integrated with pest management.

## Simple Summary

In Thailand, we studied the wild silkmoth *Cricula trifenestrata* Helfer, known for its golden silk, on cinnamon trees as a novel host plant. Our research uncovered remarkable behavioral adaptations: caterpillars exhibit sophisticated social behaviors including density-dependent egg-laying strategies and complex biphasic aggregation patterns throughout their development. Notably, 85.1% of individuals were observed to cluster together to create individual cocoons within large communal groups (ranging from 2 to over 100 cocoons), suggesting gregarious cocooning behavior with unique net-like architectural cocoon structures. By comparing four different rearing conditions, we found that laboratory-reared and hog-plum-origin caterpillars produced significantly higher quality silk cocoons (cocoon shell ratio: 5.02–5.30% vs. 3.56–3.92% in field conditions). The substantial yield from outbreak populations (>2 kg fresh weight) demonstrates considerable commercial potential. This knowledge provides crucial biological insights for conservation efforts, sustainable sericulture development, and integrated pest management strategies in the region.

## 1. Introduction

*Cricula trifenestrata* Helfer (Lepidoptera: Saturniidae), the Cricula wild silkmoth, is widely distributed across South and Southeast Asia. This species exhibits significant ecological and economic importance, embodying a paradoxical dual role as both an agricultural pest and a source of valuable biomaterials. This duality has stimulated growing interest in pest-to-product transformation strategies for sustainable development initiatives [1,2].

Ecologically, *C. trifenestrata* Helfer functions as a polyphagous defoliator capable of inflicting severe damage to forest and agricultural systems [3], with documented pest status on mango, cashew, cardamom, and cinnamon across multiple countries [4]. However, this same ecological adaptability—the ability to successfully exploit diverse host plants—underlies its considerable economic potential. The species’ robust feeding behavior and rapid development enable it to produce substantial biomass in the form of distinctive golden cocoons exhibiting valuable properties including water and bacterial resistance, rendering them highly sought after in specialized textile applications [4,5]. This pest-to-product transformation exemplifies how agricultural challenges can be converted into commercial opportunities: silk-derived proteins demonstrate health benefits including cholesterol regulation and antioxidant activity [4], while the pupae serve as a high-protein food source throughout many regions, including Thailand [6]. These multifaceted applications have catalyzed conservation efforts and sustainable rearing program development, following successful models established in Indonesia [1].

In Thailand, the ecological range of *C. trifenestrata* Helfer expanded significantly with the first documented occurrence on cinnamon (*Cinnamomum* spp.) in Chaiyaphum Province [7]. Subsequent to this initial discovery, a substantial population resurgence was observed on cinnamon trees in September 2024. While our primary focus was to document the complete life cycle on cinnamon as a novel host, an opportunistic mass outbreak on the adjacent hog plum tree provided valuable comparative data. During field investigations of this phenomenon, numerous fifth-instar larvae were observed descending en masse from an adjacent hog plum (*Spondias pinnata*) tree, revealing complex dual-host utilization patterns within the local population. While hog plum has been previously documented as a host plant in other regions of Thailand, comprehensive studies of this host plant interaction in Chaiyaphum Province remain limited [2]. Although recent research has characterized the external morphology of larvae from this population [8], and life cycle studies have been conducted on alternative hosts [2,9], comprehensive documentation of life history plasticity and gregarious cocooning behavior specifically on cinnamon as the primary novel host plant represents a significant knowledge gap. Addressing this gap is essential, as life history traits and cocoon characteristics exhibit strong host plant dependency [4]. The 2024 population resurgence provided a unique opportunity to address these knowledge gaps through the following objectives: (1) to document the complete life cycle of *C. trifenestrata* Helfer on cinnamon across field, semi-natural, and laboratory conditions to assess its life history plasticity; (2) to characterize its gregarious behaviors, specifically larval aggregation and communal cocooning, within this dual-host population; and (3) to evaluate and compare key cocoon characteristics (e.g., cocoon weight, shell weight, and pupal weight) between larvae reared on cinnamon (a novel host plant) and hog plum (a reported host plant).

## 2. Materials and Methods

### 2.1. Study Sites and Host Plant Species

**Figure 1:**
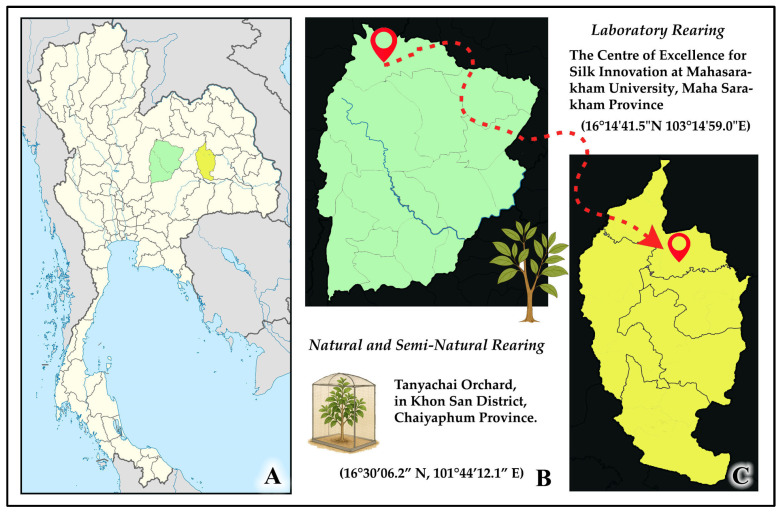
Geographic location of the study sites. (**A**) Map of Thailand indicating the locations of Chaiyaphum Province (field site) and Maha Sarakham Province (laboratory site). (**B**) The field site, Tanyachai Orchard, in Khon San District, Chaiyaphum. (**C**) The location of the Centre of Excellence for Silk Innovation at Mahasarakham University, Maha Sarakham. Base maps from Wikipedia Commons: “Chaiyaphum Province” and “Thailand_Maha_Sarakham_location_map.svg,” accessed 15 December 2024.

Field investigations and associated experiments were conducted between September and December 2024 at Tanyachai Orchard, located in Thung Luilai Sub-district, Khon San District, Chaiyaphum Province, Thailand (16°30′06.2″ N, 101°44′12.1″ E, approximately 575 m above sea level) (Figure 1). The study site comprises a mixed-fruit cultivation system with diverse plant species. The principal host plant for systematic life cycle monitoring was identified as a mature cinnamon tree (*Cinnamomum* spp.), whose taxonomic identification was previously established [7]. During the 2024 study period, an additional host plant, a mature hog plum (*S. pinnata*) tree, was identified through opportunistic field observations. Laboratory-based experimental procedures were conducted at the Centre of Excellence for Silk Innovation, Mahasarakham University.

### 2.2. Insect Material and Rearing Conditions

#### 2.2.1. Field Population Monitoring

**Figure 2:**
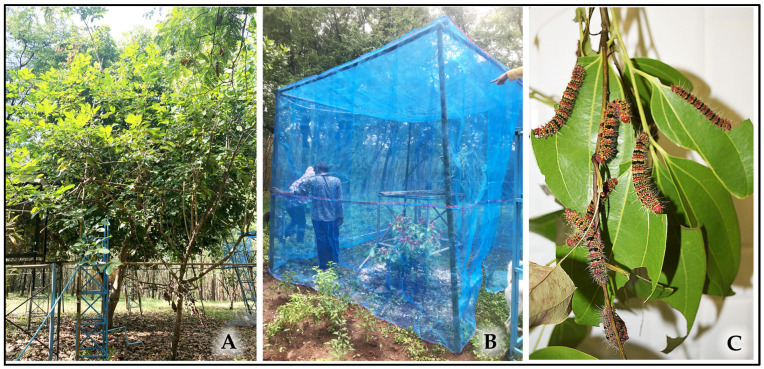
Experimental setups for rearing *C. trifenestrata* Helfer. (**A**) The mature cinnamon tree in the mixed-fruit orchard that served as the host for the wild population (field observation). (**B**) The semi-natural rearing cage (2 × 2 × 2 m) enclosing a young cinnamon tree. (**C**) A representative laboratory rearing container with young-instar larvae feeding on cinnamon leaves.

Systematic monitoring of the whole life cycle was focused on the population inhabiting the cinnamon tree (Figure 2A). A subsample of *n* = 10 larvae was randomly selected and monitored individually for precise developmental timing calculations. Concurrently, opportunistic observations were made on the nearby hog plum tree, focused on a mass dispersal event of late 5th-instar larvae.

#### 2.2.2. Semi-Natural Rearing on Cinnamon

A semi-natural experiment was established in a nylon mesh field cage (100-mesh, 2 × 2 × 2 m) erected over a young cinnamon tree (Figure 2B). A cohort of 30 wild-collected 2nd-instar larvae was introduced into the cage and fed ad libitum. From this cohort, *n* = 10 individuals were randomly selected for detailed developmental monitoring.

#### 2.2.3. Laboratory Rearing

A laboratory colony was established from wild-collected 5th-instar larvae (*n* ≈ 50) to obtain an F_1_ generation. These F_1_-generation larvae were group-reared under controlled conditions (27 ± 1 °C, 70 ± 5% RH, 12L/12D photoperiod) (Figure 2C) and initially fed exclusively on fresh cinnamon leaves. From an initial cohort of approximately 50 larvae, 28 survived to the pre-pupal stage. A subsample of *n* = 10 individuals was selected for detailed life cycle monitoring.

During the late 5th instar, a logistical challenge arose: due to the high consumption rate of mature larvae, the supply of fresh cinnamon leaves, transported from the distant field site, became insufficient. To prevent starvation, the diet was supplemented with fresh avocado (*Persea americana*) leaves, a known alternative host plant. Consequently, this laboratory cohort must be considered a mixed-diet group (cinnamon supplemented with avocado) during its final feeding stage. For pupation, larvae were allowed to construct cocoons on these same potted avocado trees.

#### 2.2.4. Opportunistic Collection from Hog Plum Trees

This cohort was not part of the primary study design, which focused exclusively on cinnamon as a novel host plant. Instead, it represented an opportunistic collection when a natural outbreak was unexpectedly observed on a mature hog plum (*S. pinnata*) tree adjacent to the study site during the field investigation period. The mass dispersal of late 5th-instar larvae from this tree was collected and consolidated into large holding cages for comparative purposes. These larvae were provided with fresh cinnamon leaves. While most larvae were in the pre-pupal wandering stage and began cocooning almost immediately, a notable portion actively fed on the supplied cinnamon leaves for several days before initiating pupation. Consequently, this cohort represents a group with a primary hog plum diet, supplemented with cinnamon for some individuals in the final instar. Following the mass pupation event, a random subsample of cocoons was selected from the bulk harvest for detailed trait analysis, without distinguishing between larvae that did or did not feed on the supplemental cinnamon. This opportunistic collection provided valuable comparative data on cocoon quality between the novel host plant (cinnamon) and a previously documented host plant (hog plum).

### 2.3. Data Collection Protocols

#### 2.3.1. Life History and Behavioral Documentation

Laboratory cohort developmental stages were recorded at 24 h intervals throughout the complete life cycle. Natural field and semi-natural caged cohorts were monitored at 48 h intervals during active larval feeding phases, with increased frequency to daily observations during critical developmental transitions, including molting events and pupation initiation. Behavioral patterns and developmental milestones were systematically documented through detailed field notes and photographic records.

For each experimental condition, developmental duration data were collected from *n* = 10 randomly selected individuals to calculate mean ± standard deviation for statistical analysis. For the field cohort, which originated from a single egg batch, developmental timing for highly synchronous early instars (1–3) was recorded for the group as a whole, while late instars (4–5) were monitored individually.

#### 2.3.2. Oviposition Pattern Analysis

Quantitative assessment of oviposition behavioral plasticity was conducted through systematic analysis of naturally occurring egg deposition patterns. Five naturally deposited egg batches on cinnamon foliage were examined in field conditions to determine the frequency and characteristics of linear ‘string-of-beads’ oviposition arrangements. Batch sizes and spatial arrangements were recorded for each observation.

For comparative analysis under high-density laboratory conditions, 15 egg clusters were randomly sampled from over 30 observed clusters throughout the laboratory setup. These samples represented diverse substrate types (including leaf surfaces and artificial container surfaces) to quantify the occurrence of clustered and multi-layered oviposition patterns. Based on our sample, clusters were grouped into three size categories for analysis: small (1–7 eggs), medium (11–30 eggs), and large (60+ eggs). Each cluster was categorized by size and substrate type to establish frequency distributions.

#### 2.3.3. Gregarious Cocooning Behavior Assessment

The frequency of occurrence and cluster composition (number of individual cocoons per aggregation) of gregarious cocooning behavior was systematically recorded across three primary experimental cohorts (natural field, semi-natural caged, and laboratory populations). A total of 47 cocooning sites were analyzed across natural field (*n* = 30), semi-natural caged (*n* = 7), and laboratory (*n* = 10) conditions to provide a quantitative assessment of social cocooning behavior under varying environmental conditions and population density parameters.

Each cocooning site was classified into size categories: solitary (1 cocoon), small (2–10 cocoons), medium (11–30 cocoons), and large (31–100+ cocoons) aggregations. The hog plum cohort was observed separately under contained conditions but excluded from the comparative cocooning behavior analysis due to spatial constraints imposed by large holding containers.

#### 2.3.4. Morphometric Cocoon Analysis

A randomized subsample consisting of five male and five female cocoons was selected from each of the four experimental cohorts (total *n* = 40) for comprehensive comparative morphometric analysis. Sex determination was based on pupal morphology, specifically the presence of a fine longitudinal line on the ventral side of the 8th abdominal segment in females, which is absent in males (as described in [7]). Three primary quantitative traits were measured for each specimen: fresh cocoon mass (g), pupal mass (g), and cocoon shell mass (g). The cocoon shell ratio (CSR, %) was subsequently calculated using the standardized formula: CSR = (shell mass/total cocoon mass) × 100.

### 2.4. Statistical Analysis

All quantitative data are presented as mean ± standard deviation (SD). Developmental duration comparisons among the three cinnamon-reared cohorts (natural field, semi-natural caged, and laboratory treatments) were analyzed using one-way ANOVA followed by Games–Howell post hoc tests due to unequal variances. Cocoon morphometric characteristics (mass measurements and CSR values) among the four experimental cohorts were analyzed using Welch’s ANOVA due to heterogeneity of variances, followed by Tukey HSD post hoc tests for multiple pairwise comparisons where homogeneity assumptions were met.

Gregarious cocooning cluster sizes among the three primary experimental cohorts were analyzed using descriptive statistics to determine frequency distributions across the defined size categories. Oviposition behavioral patterns were analyzed through descriptive statistical methods, calculating frequency distributions for each observed pattern type under field and laboratory conditions. Statistical significance was established at α = 0.05 for all analyses. All statistical procedures were performed using SPSS statistical software (version 20).

## 3. Results

### 3.1. Life Cycle Duration Under Different Rearing Conditions

A one-way analysis of variance (ANOVA) revealed that the rearing environment had a statistically significant effect on the total life cycle duration of *C. trifenestrata* Helfer fed on cinnamon (F_2,27_ = 16.838, *p* < 0.001, η^2^ = 0.555). Post hoc analysis using the Games–Howell test confirmed that the laboratory-reared cohort exhibited the longest mean life cycle duration (62.30 ± 3.68 days), which was significantly longer than both the natural field (56.30 ± 1.83 days) and semi-natural caged (56.90 ± 1.60 days) cohorts (pairwise comparisons, *p* < 0.001). No significant difference was detected between the natural field and semi-natural caged conditions (*p* = 0.859). These results are summarized in Table 1.

### 3.2. Stage-Specific Analysis

This prolonged development in the laboratory was attributable to three key stages:

Egg Stage: The incubation period in the laboratory (11.00 ± 0.67 days) was significantly longer than in the natural field (9.50 ± 0.53 days) and semi-natural caged (9.40 ± 0.52 days) conditions (F_2,27_ = 24.371, *p* < 0.001, η^2^ = 0.644).

Pupal Stage: The duration of the pupal stage in the laboratory (13.70 ± 1.42 days) was significantly longer than that of the natural field (11.10 ± 0.88 days) and semi-natural caged (11.20 ± 1.03 days) cohorts (F_2,27_ = 16.934, *p* < 0.001, η^2^ = 0.556).

Adult Stage: Adult longevity was significantly greater in the laboratory (5.20 ± 0.42 days) compared to the semi-natural caged condition (4.60 ± 0.52 days) (F_2,27_ = 4.574, *p* = 0.019, η^2^ = 0.253).

In contrast, larval development showed remarkable consistency across rearing environments. None of the five larval instars exhibited significant duration differences among the three treatments (*p* > 0.05 for all stages). Following the final larval instar, the cocoon spinning stage was consistently observed to last for one day across all three rearing conditions before pupation commenced.

### 3.3. Morphological Characteristics and Developmental Stages

**Figure 3:**
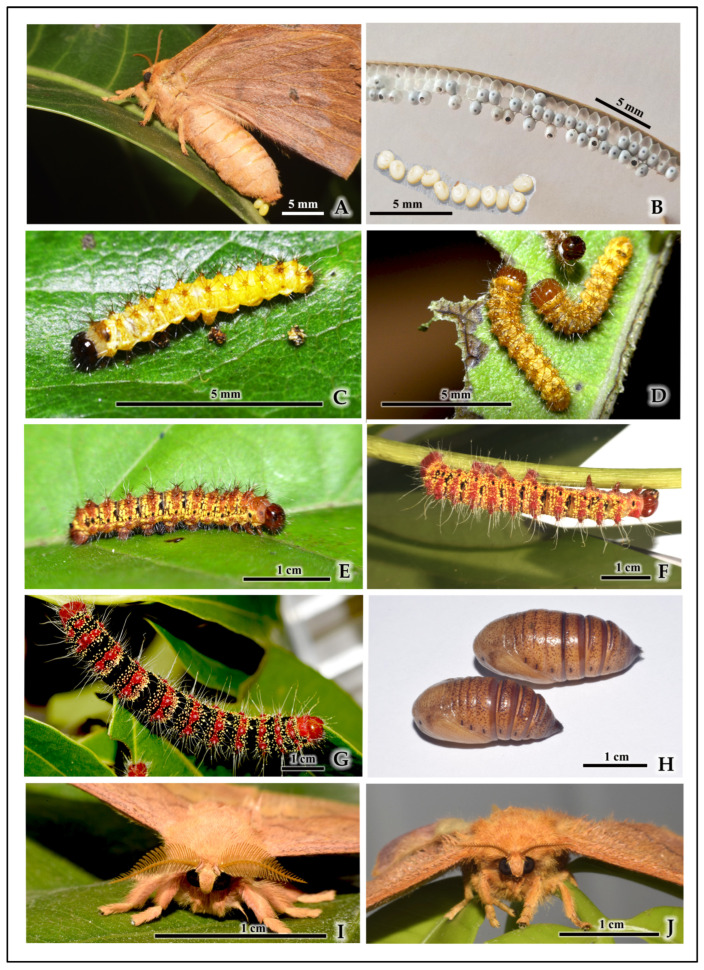
Life stages and key morphological features of *C. trifenestrata* Helfer. (**A**) Adult female depositing eggs (scale bar = 5 mm). (**B**) Egg string showing individual eggs laid in a linear arrangement (scale bar = 5 mm). (**C**) First-instar larva (scale bar = 5 mm). (**D**) Second-instar larva (scale bar = 5 mm). (**E**) Third-instar larva (scale bar = 1 cm). (**F**) Fourth-instar larva (scale bar = 1 cm). (**G**) Fifth-instar larva (scale bar = 1 cm). (**H**) Pupa (dorsal and ventral views) (scale bar = 1 cm). (**I**) Adult male (showing quadripectinate antennae) (scale bar = 1 cm). (**J**) Adult female (showing bipectinate antennae) (scale bar = 1 cm).

*C. trifenestrata* Helfer undergoes five larval instars before pupation. The transition between each instar is confirmed by the process of ecdysis (molting), where the larva becomes immobile and sheds its old exoskeleton to emerge into the next stage. Key diagnostic features for identifying each life stage are summarized in Table 2, with representative images presented in Figure 3.

### 3.4. Behavioral Observations

#### 3.4.1. Density-Dependent Oviposition Plasticity

**Figure 4:**
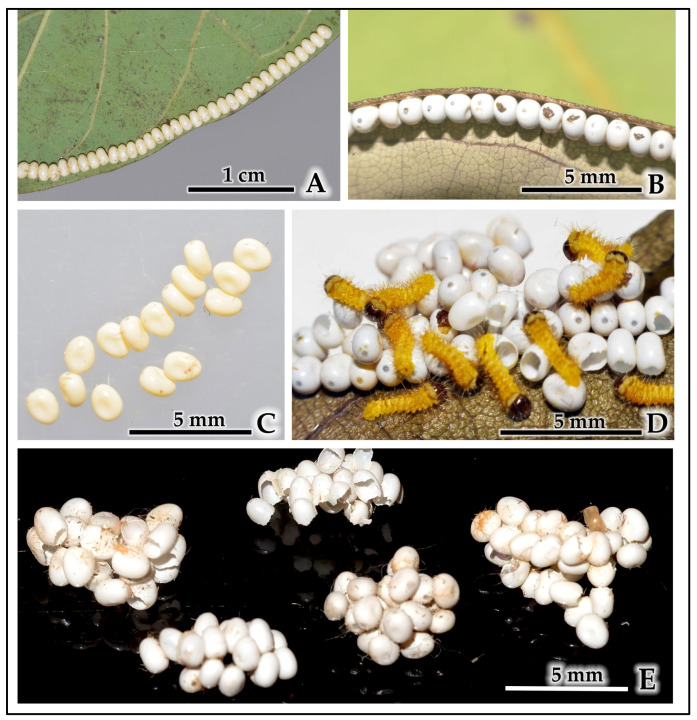
Oviposition plasticity in *C. trifenestrata* Helfer. (**A**) Linear arrangement of eggs on a leaf margin in the field (scale bar = 1 cm). (**B**) Close-up view of eggs laid in a linear arrangement on a leaf margin in the field (scale bar = 5 mm). (**C**) Clustered eggs from a laboratory rearing, showing a disorganized arrangement and variable sizes (scale bar = 5 mm). (**D**) Newly hatched larvae emerging from a laboratory egg cluster, illustrating the dense and irregular egg mass (scale bar = 5 mm). (**E**) Multiple egg clusters of varying sizes from laboratory conditions on black background, demonstrating the range of cluster formations (scale bar = 5 mm).

Field oviposition patterns: In natural field conditions, females consistently exhibited organized egg-laying behavior. Eggs were laid exclusively in single-file lines (1–3 rows) along leaf margins, resembling a “string-of-beads” arrangement (Figure 4A), with close-up details showing the precise linear organization (Figure 4B). Analysis of five naturally deposited egg batches on cinnamon foliage showed exclusively linear arrangements with batch sizes of 34, 135, 169, 180, and 260 eggs per batch (mean = 155.6 ± 84.9 eggs per batch, *n* = 5). All field observations (100%, *n* = 5) followed this organized, edge-specific oviposition pattern on host plant leaves only (Table 3).

Laboratory oviposition patterns: Under controlled laboratory conditions with higher conspecific density, females exhibited markedly different oviposition behavior. Instead of organized linear arrangements, eggs were laid in dense, multi-layered clusters on diverse substrates including host plant leaves, laboratory container floors, aluminum trays, and mesh netting (Figure 4C). These clustered eggs often resulted in newly hatched larvae emerging simultaneously from the dense egg masses (Figure 4D). Analysis of 15 egg clusters randomly sampled from over 30 observed clusters throughout the laboratory setup revealed substantial size variation (Figure 4E): small clusters (1–7 eggs: 46.7% frequency, *n* = 7), medium clusters (11–30 eggs: 40.0% frequency, *n* = 6), and large aggregations (60+ eggs: 13.3% frequency, *n* = 2). No intermediate-sized clusters (8–10 or 31–59 eggs) were found in our random sample of 15 clusters, though this may reflect the limited sample size rather than a true bimodal distribution in the population.

Statistical comparison: The shift from 100% linear arrangements in field conditions to 100% clustered formations in laboratory settings demonstrates significant density-dependent behavioral plasticity (χ^2^ = 15.0, df = 1, *p* < 0.001). Laboratory conditions resulted in opportunistic substrate utilization across multiple surface types compared to exclusive host plant oviposition in field conditions (Table 3).

#### 3.4.2. Larval Aggregation and Gregarious Cocooning Behavior

A dynamic, three-phase pattern of aggregation and dispersal was observed throughout the larval stages, with collective behavior originating from siblings hatching synchronously from an egg batch on a single leaf (see Supplementary Videos S1–S3 for detailed behavioral documentation).

Phase 1: Early Sibling Groups (First–Third instars)

**Figure 5:**
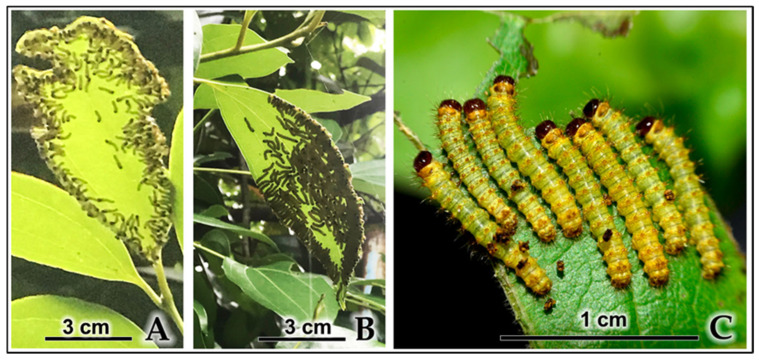
Gregarious feeding by early-instar *C. trifenestrata* Helfer larvae. (**A**) Early-instar larvae exhibiting collective feeding behavior in a natural field setting (scale bar = 3 cm). (**B**) Early-instar larvae forming dense clusters on the underside of a host plant leaf in a natural field setting (scale bar = 3 cm). (**C**) A close-up of a larval group under laboratory conditions, showing detailed morphology of the individual caterpillars (scale bar = 1 cm).

Newly hatched larvae consistently remained in close proximity on their natal leaf, feeding and molting in synchronized cohorts (Figure 5A). Multiple lines of evidence from both field observations and controlled laboratory experiments demonstrate active gregarious behavior extending beyond passive proximity:

Field observations: Quantitative analysis of photographic documentation revealed that first-instar larvae maintained extremely dense feeding aggregations (173 ± 31 individuals per leaf; range 133–225; *n* = 5 leaves counted from Figure 5A,B and Video S1) even when numerous other suitable leaves were available nearby on the same plant or adjacent plants. Figure 5C shows a close-up view of a smaller laboratory group (eight individuals), demonstrating the detailed morphology of aggregating larvae. Video documentation (Video S1) shows the complete developmental pattern, with third-instar larvae forming medium-sized groups (10–12 individuals per leaf) and fifth-instar larvae dispersing into small groups (3–5 individuals across adjacent branches) or feeding solitarily (1 individual per leaf).

**Figure 6:**
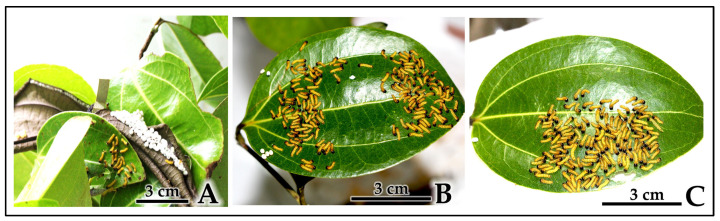
Controlled re-aggregation experiments demonstrating active gregarious behavior in 1st-instar *C. trifenestrata* Helfer larvae. (**A**) Manual transfer showing initial scattered distribution immediately after placement (scale bar = 3 cm). (**B**) Temporary exploration phase with larvae separating into two groups around leaf perimeter (scale bar = 3 cm). (**C**) Complete re-aggregation into single feeding cluster within 30 min, demonstrating preference for group feeding over individual foraging (scale bar = 3 cm).

Controlled relocation experiments: When first-instar larvae were individually transferred using fine brushes to fresh host plants with abundant leaf area, scattered individuals consistently re-aggregated within 30 min to 2 h, forming dense feeding clusters on single leaves despite ample feeding space across multiple leaves (Figure 5B and Figure 6A–C). This re-aggregation pattern was observed across five replicate trials (*n* = 5, 100% success rate), with each trial involving 80–100 larvae that initially explored and temporarily separated into multiple smaller groups before ultimately converging into cohesive feeding aggregations.

**Figure 7:**
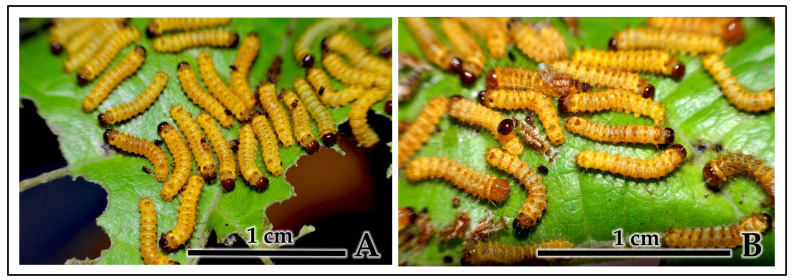
Mass synchronous molting behavior in *C. trifenestrata* Helfer larvae. (**A**) First-instar larvae entering molting phase with immobilized postures in close proximity (scale bar = 1 cm). (**B**) Active molting process showing characteristic brown head capsules (old exuviae) being shed during 1st-to-2nd-instar transition, demonstrating coordinated ecdysis and group cohesion during vulnerable developmental phase (scale bar = 1 cm).

Synchronized molting behavior: During molting periods, larvae exhibited specific observable behaviors supporting coordinated ecdysis (Figure 7A,B). First, pre-molt larvae ceased feeding simultaneously and maintained close physical contact while adopting immobilized postures (Figure 7A). Second, examination of molting aggregations revealed concentrated deposits of shed exuviae and head capsules within larval groups, with multiple cast skins visible in confined areas rather than scattered throughout available space (Figure 7B). This spatial concentration of molt remnants, combined with the maintenance of group cohesion throughout ecdysis, provides evidence of temporal synchronization rather than asynchronous individual molting events.

**Figure 8:**
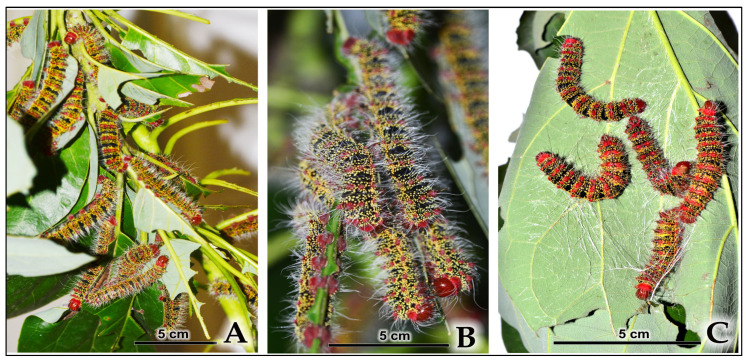
Aggregation of late-instar *C. trifenestrata* Helfer larvae in the laboratory. (**A**) Larvae grouping and feeding on host plant foliage (scale bar = 5 cm). (**B**) A close-up view of a dense aggregation of late-instar larvae on host plant branch tip (scale bar = 5 cm). (**C**) A pre-pupal aggregation of final-instar larvae on a single leaf immediately prior to the commencement of aggregated cocooning site selection, where each individual spins its own cocoon within the cluster (scale bar = 5 cm).

**Figure 9:**
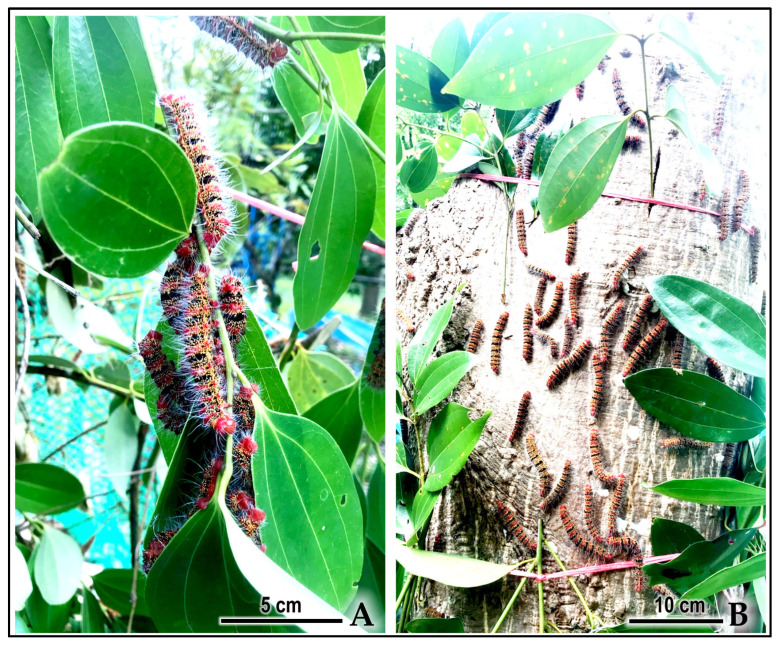
Gregarious feeding and pre-pupal aggregation of last-instar *C. trifenestrata* Helfer larvae on a cinnamon tree under natural field conditions. (**A**) A characteristic feeding aggregation of late-instar larvae on the host plant leaves (scale bar = 5 cm). (**B**) Larvae migrating down the trunk of the tree to form a pre-pupal aggregation site at the base for the construction of a cocoon cluster (scale bar = 10 cm).

Phase 2: Gradual Dispersal (Fourth–Fifth Instars) The tight aggregations observed in early instars gradually dispersed during the fourth instar, as primary groups fissioned into smaller subgroups. By the fifth and final instar, larvae were observed feeding largely in isolation or in small groups of three to four individuals (Video S2) (Figure 8A and Figure 9A). This transition coincided with increased individual body size and corresponding resource requirements. Laboratory observations showed that even when dispersed, late-instar larvae maintained some degree of aggregation on branch tips (Figure 8B).

Phase 3: Pre-Pupal Re-Aggregation and Gregarious Cocooning

After their final feeding period, mature larvae exhibited renewed aggregation behavior for gregarious cocooning, where larvae actively aggregate at shared sites to construct individual cocoons within communal clusters. In laboratory conditions, pre-pupal aggregations of final-instar larvae were observed clustering on single leaves immediately before initiating cocooning site selection (Figure 8C). Multiple lines of evidence demonstrate active aggregation independent of environmental funneling effects (Video S3).

**Figure 10:**
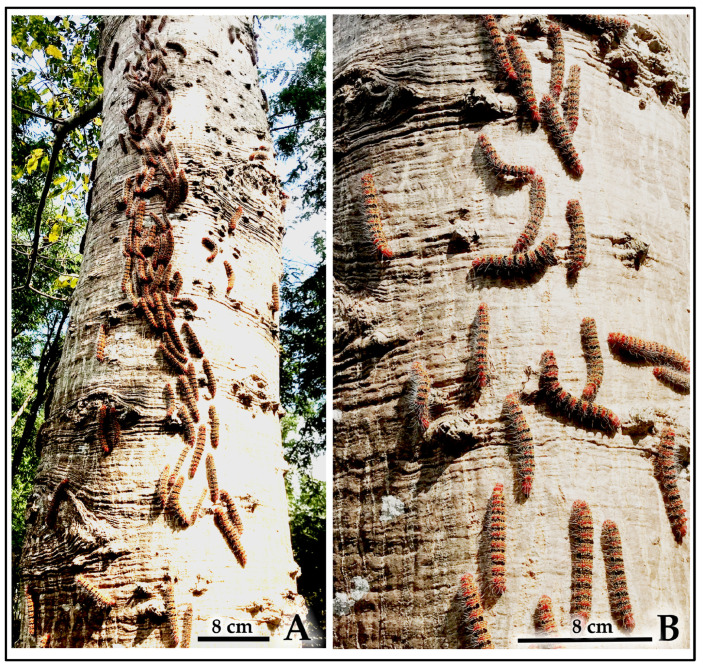
Mass downward migration of 5th-instar *C. trifenestrata* Helfer larvae from a hog plum tree to the ground. (**A**) Overall view of 5th-instar larvae descending the trunk of a hog plum tree to aggregate (scale bar = 8 cm). (**B**) Close-up view of a dense aggregation of 5th-instar larvae migrating down the trunk of a hog plum tree (scale bar = 8 cm).

Diverse aggregation site formation: Field observations documented cocoon clusters at multiple locations including tree bases, beneath leaves on host trees, and on adjacent vegetation. Pre-pupal larvae were observed at these sites forming clusters with cocoons positioned in direct contact or close proximity to each other (Figure 9B and Figure 10A,B). Larvae moved from upper feeding areas to lower aggregation sites where cluster formation occurred.

**Figure 11:**
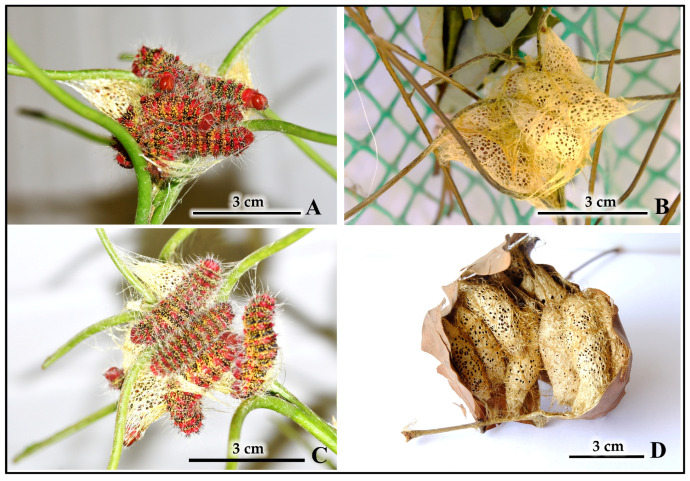
The process of cocoon cluster formation through gregarious cocooning by *C. trifenestrata* Helfer larvae. (**A**) Early aggregation of final-instar larvae initiating a shared silk matrix that forms the foundational framework of the cocoon cluster (scale bar = 3 cm). (**B**) Completed fresh cocoon cluster, showing its characteristic reticulated (net-like) structure (scale bar = 3 cm). (**C**) Dense aggregation of final-instar larvae collectively spinning an extensive silk matrix, reinforcing the developing cocoon cluster (scale bar = 3 cm). (**D**) Mature, weathered cocoon cluster attached to a dried leaf, with a section removed to reveal the individual pupal cells within (scale bar = 3 cm).

Early aggregations of final-instar larvae initiate a shared silk matrix that forms the foundational framework of the cocoon cluster (Figure 11A). As cocooning progresses, dense aggregations of larvae collectively spin an extensive silk matrix, reinforcing the developing cluster structure (Figure 11C). The completed fresh cocoon clusters display a characteristic reticulated (net-like) structure (Figure 11B), which eventually mature into weathered clusters that, when sectioned, reveal individual pupal cells within the communal framework (Figure 11D).

Laboratory evidence of clustering behavior: In controlled settings with distributed host plants, video documentation (Video S2) showed pre-pupal larvae forming cocoon clusters rather than pupating in isolation. Larvae were observed moving between plants and subsequently pupating at sites where other individuals were already present or arriving, resulting in grouped cocoons rather than scattered individual cocoons.

Spatial patterns during aggregation: Video recordings (Video S2) captured multiple larvae moving along similar routes when transitioning from feeding to pupation areas. Sequential observations showed different individuals using the same pathways, with larvae ultimately forming clusters at specific sites rather than dispersing throughout available space.

#### 3.4.3. Quantitative Analysis of Gregarious Cocooning Behavior

**Figure 12:**
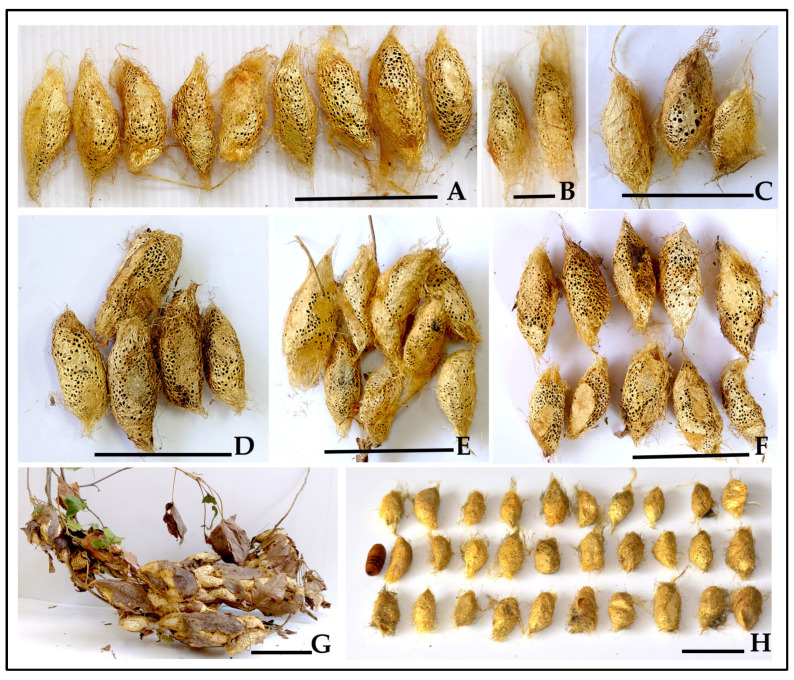
Cocoon cluster size distribution in *C. trifenestrata* Helfer. (**A**) Solitary cocoons (14.9% frequency) (scale bar = 5 cm). (**B**) Small clusters (2–3 cocoons, 48.9% frequency) (scale bar = 3 cm). (**C**) Small medium clusters (3–5 cocoons) (scale bar = 3 cm). (**D**) Medium cluster (8–10 cocoons) (scale bar = 5 cm). (**E**) Medium cluster (≈10 cocoons) in circular formation (scale bar = 5 cm). (**F**) Large medium cluster (15+ cocoons) (scale bar = 5 cm). (**G**) Large cluster in field conditions (31–100+ cocoons, 17.0% frequency) (scale bar = 8 cm). (**H**) Individual cocoons extracted from single cluster (34 total) showing individual cocoon structure and pedunculate stalks, demonstrating that each larva constructs its own separate cocoon within the aggregated site (scale bar = 5 cm).

Systematic recording of all cocooning sites across the three primary experimental cohorts (natural field, semi-natural caged, and laboratory populations) revealed that gregarious cocooning was the dominant strategy, with individual larvae constructing separate cocoons within shared communal sites. For this study, an “aggregated cocoon cluster” was defined as two or more individual cocoons positioned in direct contact or close proximity to each other (Figure 12B–G), while a “solitary cocoon” refers to a single isolated cocoon (Figure 12A). From 47 total cocooning sites analyzed, 85.1% formed aggregated cocoon clusters (40 out of 47 sites, ranging from 2 to >100 cocoons per cluster), while 14.9% were solitary cocoons (7 out of 47 sites). Figure 12H demonstrates that each larva constructs its own individual cocoon within these clusters, as evidenced by 34 separate cocoons extracted from a single aggregation site. The frequency distribution of cocoon cluster sizes varied across environmental conditions (Table 4).

Small clusters (2–10 cocoons) were most common overall (48.9%), while large clusters (31–100+ cocoons) were exclusively observed in natural field conditions (17.0%), suggesting that environmental space and population density influence maximum cluster sizes. The consistent formation of aggregated cocoons across all three environmental conditions provides strong evidence for intrinsic gregarious cocooning behavior (Figure 12).

### 3.5. Cocoon Quality and Commercial Potential

#### 3.5.1. Rearing Environment Effects on Cocoon Characteristics

The results of Welch’s ANOVA revealed that rearing conditions had statistically significant effects on several key cocoon traits (Table 5). For female cocoons, significant differences were found in cocoon shell weight (Welch’s F_3,16_ = 5.230, *p* = 0.027) and CSR (Welch’s F_3,16_ = 13.172, *p* = 0.002). For male cocoons, a significant difference was also found in the cocoon shell ratio (Welch’s F_3,16_ = 4.495, *p* = 0.038).

Post hoc analysis using Tukey’s HSD test indicated that the mean CSR for laboratory and hog plum cohorts was significantly higher than that of natural field and semi-natural caged cohorts. Furthermore, the mean cocoon shell weight of females from the hog plum cohort was significantly greater than that of the semi-natural caged cohort.

#### 3.5.2. Mass Production Potential from Opportunistic Hog Plum Collection

Cluster formation frequency under contained conditions: Observations from the opportunistic mass collection of hog plum larvae provided additional insights into gregarious cocooning behavior under contained conditions. When pre-pupal larvae from the natural hog plum outbreak were transferred to large holding cages and provided with fresh cinnamon leaves, the frequency of cluster formation was remarkably high, with virtually all individuals (>95%, estimated from bulk observation) forming aggregated cocoons rather than solitary ones. This rate was higher than the 85.1% aggregation rate observed under natural field conditions, though direct statistical comparison was not possible due to the different experimental setup.

**Figure 13:**
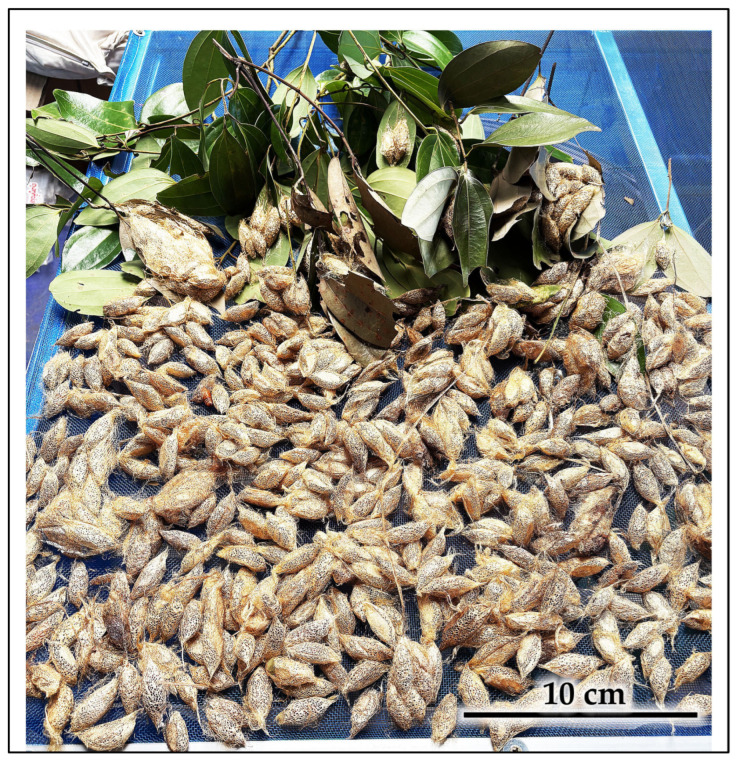
A representative portion of the bulk cocoon harvest obtained from the opportunistic rearing of the hog-plum-derived cohort. Larvae were provided with cinnamon leaves in captivity until pupation.

**Figure 14:**
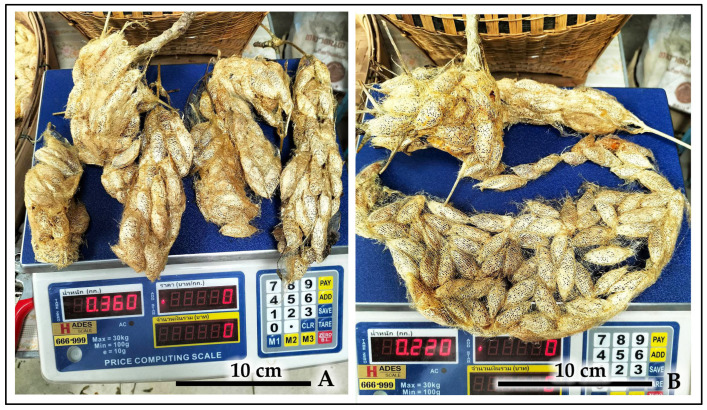
Examples of communal cocoon clusters from the hog-plum-derived cohort, illustrating their structure and harvest weight. (**A**) Several elongated clusters with a combined weight of 0.360 kg. (**B**) A single, large, basket-shaped communal cocoon cluster weighing 0.220 kg.

Quantitative productivity analysis: The opportunistic mass collection from the hog plum outbreak yielded a substantial bulk harvest with a total fresh weight exceeding 2.0 kg. This large yield consisted predominantly of communal cocoon clusters, with cluster sizes ranging from small groups (2–5 cocoons) to large aggregations (50+ cocoons) (Figure 13 and Figure 14). Individual large clusters reached weights of 0.220 kg fresh weight, demonstrating the practical efficiency of gregarious cocooning behavior for bulk silk production under managed conditions.

Commercial implications: This high productivity, combined with the superior silk quality from certain rearing conditions (laboratory and hog plum showing significantly higher cocoon shell ratios), indicates substantial potential for both pest management and silk production applications. The ability to achieve high silk yields (>2.0 kg) from contained systems demonstrates the practical feasibility of developing sustainable sericulture systems based on *C. trifenestrata* Helfer as a supplementary host to cinnamon.

## 4. Discussion

### 4.1. Developmental Plasticity and Environmental Adaptation

*C. trifenestrata* Helfer demonstrated remarkable phenotypic plasticity across rearing environments. Laboratory development (62.30 ± 3.68 days) significantly exceeded field (56.30 ± 1.83 days) and semi-natural caged conditions (56.90 ± 1.60 days; F_2,27_ = 16.838, *p* < 0.001), aligning with the 60–170 day range reported across different hosts [4,9,10]. Extended egg stages (11.00 ± 0.67 vs. 9.50 ± 0.53 days) and pupal stages (13.70 ± 1.42 vs. 11.10 ± 0.88 days) in laboratory conditions (*p* < 0.001) may reflect nutritional constraints of excised foliage [11]. Notably, faster second-instar development under laboratory conditions suggests early instars may benefit from predator-free environments despite compromised food quality, indicating adaptive trade-offs between nutrition and safety.

### 4.2. Novel Host Adaptation and Dual-Host Strategy

This study provides the first evidence of complete *C. trifenestrata* Helfer development on cinnamon (*Cinnamomum* spp.), a novel host plant, in Thailand, revealing a previously undocumented dual-host utilization pattern. The 40% larval size reduction on cinnamon (7.5 ± 0.10 cm vs. 13.5–14.0 cm on traditional hosts) indicates suboptimal nutrition [4], yet normal color progression and behavioral patterns suggest adaptive plasticity rather than stress responses. The observed foraging strategy—larvae initially feeding on hog plum (*S. pinnata*) before dispersing to adjacent cinnamon—may represent a novel resource exploitation mechanism. This flexibility could enhance population persistence when primary hosts become depleted, suggesting the ecological adaptability enabling this species to function as both an agricultural pest and a valuable bioresource.

### 4.3. Social Behavior and Collective Decision-Making

#### 4.3.1. Implications of Density-Dependent Oviposition Plasticity

Oviposition behavior exhibited density-dependent plasticity that may optimize offspring survival. Field conditions produced linear “string-of-beads” patterns (155.6 ± 84.9 eggs/batch, *n* = 5), while laboratory crowding triggered clustered deposition (46.7% small, 40.0% medium, 13.3% large clusters; χ^2^ = 15.0, *p* < 0.001). This flexibility contrasts with rigid strategies in other lepidopterans [12] and parallels findings in *Eucheira socialis westwoodi*, where clumped egg masses enhanced larval coalescence and survival compared to isolated groups [13].

#### 4.3.2. Active Gregarious Behavior Throughout Development

Controlled experiments provide evidence consistent with active gregarious behavior rather than passive aggregation. First-instar larvae frequently re-aggregated into dense feeding clusters within 2–4 h when scattered (100% success rate, *n* = 5 trials), despite abundant solitary feeding opportunities. This preference for group cohesion over resource optimization may provide multiple adaptive advantages: predator dilution through selfish herd effects [14], amplified aposematic signals through signal augmentation where combined warning displays become more conspicuous and effective at deterring predators [15,16], enhanced defense through collective urticating spine display [17], improved thermoregulation, and enhanced ability to overcome plant defenses.

Group size effects are critical for survival, as demonstrated in related species where larger groups show enhanced foraging efficiency and survival rates. In *E. socialis westwoodi*, groups of 400 individuals fed more frequently (25.8% vs. 16.7%) and gained more weight (1.31 vs. 1.08 mg per capita) than smaller groups of 50, due to increased physical contact frequency stimulating feeding initiation [13].

#### 4.3.3. Ontogenetic Social Shifts and Cooperative Mechanisms

The species exhibits biphasic larval sociality, with early instars (first–third) observed in tight aggregations and late instars (fourth–fifth) typically occurring in smaller groups of 3–4 individuals or solitarily. This pattern may reflect changing cost–benefit ratios throughout development, potentially balancing resource competition against protection benefits [18]. The observed ontogenetic shift suggests adaptive behavioral flexibility in response to varying ecological pressures, though the specific mechanisms require further investigation.

Kin selection theory may explain the evolution of such gregarious behavior [19]. While high relatedness among full-sibling larvae from single egg batches could favor cooperation through inclusive fitness benefits, the maintenance of gregarious behavior, particularly during pupation, appears to be driven primarily by direct individual benefits. The observed communal silk spinning represents “by-product beneficence” [20], where individuals constructing pupal chambers for their own protection simultaneously strengthen the collective structure. This mechanism—occurring when self-serving acts inadvertently benefit others without requiring reciprocity—may provide mutual advantages including enhanced structural support, improved microclimate buffering, and potential predator deterrence that exceed individual capabilities [20].

These individual-level benefits suggest that gregarious behavior can evolve and persist through direct fitness advantages alone, without necessarily requiring inclusive fitness considerations [20,21]. While early-instar groups may comprise siblings from single egg batches, the continuation of gregarious behavior in later instars—when group composition potentially includes non-relatives—indicates that kin selection may not be essential to explain this behavior. As West et al. [21] clarify, behaviors can provide direct fitness benefits (+/+, mutual benefit) even when performed in groups containing relatives, and the distinction between direct and indirect benefits is crucial for understanding the selective forces at work. The by-product nature of the benefits allows cooperation to emerge from self-interested motivations, providing a parsimonious explanation for the observed social patterns without invoking complex recognition systems or altruistic behaviors [21].

#### 4.3.4. Gregarious Cocooning Behavior

Our observations suggest gregarious cocooning in *C. trifenestrata* Helfer, with 85.1% of individuals forming aggregated cocoons (40 of 47 sites analyzed). Several lines of evidence indicate active aggregation during pupation: (1) cocoon clusters occurred at diverse spatial locations including tree bases, branches at different heights, and adjacent vegetation; (2) pre-pupal larvae were observed traveling distances exceeding 50 cm to join existing aggregation sites while bypassing closer unoccupied areas; (3) aerial clustering on leaves and branches occurred independently of potential ground-level funneling effects; and (4) high aggregation frequency persisted across different rearing conditions, with increased aggregation under spatial constraints (>95% in laboratory vs. 85.1% in natural conditions).

Such behavior appears to be uncommon among Saturniidae, with few documented cases of communal pupation strategies. The aggregation pattern is consistent with selfish herd dynamics [14], where joining a group may reduce individual predation risk through dilution effects. The resulting collective architecture may provide enhanced protection while maintaining individual emergence independence—illustrating how simple individual decision rules could generate group-level benefits without requiring altruism or kin recognition.

### 4.4. Commercial Silk Production and Integrated Pest Management

#### 4.4.1. Silk Quality and Host-Mediated Effects

Environmental conditions influenced silk quality. Laboratory and hog-plum-origin females showed higher CSR: 5.02 ± 0.72% and 5.30 ± 0.30% compared to field conditions (3.92 ± 0.51%; Welch’s F_3,16_ = 13.172, *p* = 0.002). These CSR values suggest *C. trifenestrata*’s potential as a commercial wild silk source, positioning it among viable alternative fiber producers [4].

The persistence of elevated CSR in hog-plum-origin larvae despite post-collection cinnamon feeding may reflect host-mediated carry-over effects, possibly reflecting early-instar nutritional priming influencing silk gland development. This finding has important implications for cultivation strategies and silk quality optimization.

#### 4.4.2. Pest-to-Product Transformation Strategy

The substantial yield from hog plum outbreak populations (>2 kg fresh cocoons) suggests how pest management can generate economic value. Individual communal clusters reaching 0.220 kg enable efficient bulk harvesting that removes hundreds of potential breeding adults. This approach represents a promising pest-to-product strategy that could reduce agricultural damage while generating rural income, similar to successful programs demonstrated in Southeast Asian agricultural systems [5].

Gregarious cocooning behavior facilitates this strategy—aggregated cocoons are easily located and collected with higher efficiency than targeting dispersed individuals. Combined with the silk’s antimicrobial properties and golden coloration valued in specialty markets [1], this creates economically viable incentives for community-based integrated pest management.

### 4.5. Evolutionary Significance and Ecological Implications

The behavioral repertoire of *C. trifenestrata* Helfer represents sophisticated adaptation to variable environments through notable phenotypic plasticity. The integration of density-dependent oviposition, ontogenetic social shifts, active gregarious behaviors, and flexible host utilization may enable success across diverse habitats. Recent comparative analyses across 676 lepidopteran species demonstrate that behavioral transitions in social organization are more constrained in cryptic species, with group-living-to-solitary transitions occurring primarily in aposematic lineages [16]. The capacity for cocoon aggregations ranging from solitary to >100 individuals, mediated by active behavioral choices, suggests complex decision-making mechanisms warranting comparative investigation across Saturniidae.

This species may serve as a valuable model for understanding the evolution of social behavior in Lepidoptera, particularly the rare phenomenon of gregarious pupation. The behavioral repertoire demonstrates evolutionary lability in social organization, consistent with broader patterns where aposematic lepidopteran species show greater flexibility in aggregation tendencies compared to cryptic species [16]. The collective silk architecture emerging from coordinated construction behavior illustrates how individual self-interest may generate group benefits through by-product beneficence, a fundamental cooperative mechanism where selfish acts simultaneously benefit others without requiring reciprocal arrangements or complex recognition systems [20]. This simple form of cooperation may represent the evolutionary foundation from which more complex social behaviors develop, allowing for the maintenance of group benefits in mixed-relatedness aggregations.

### 4.6. Study Limitations and Future Research Directions

#### 4.6.1. Methodological Constraints

Several factors limit firm conclusions about host effects. The mixed laboratory diet (cinnamon supplemented with avocado) during the late fifth instar may have influenced silk production metrics, while opportunistic sampling from hog plum prevented controlled comparisons. Small sample sizes (*n* = 5 per sex per treatment) limited statistical power for detecting subtle host effects, and the absence of long-term population monitoring restricts understanding of demographic consequences.

#### 4.6.2. Priority Research Areas

Future investigations should address seven critical areas: (1) chemical ecology of trail pheromones mediating aggregation behavior, (2) kin recognition mechanisms underlying sibling aggregation preferences, as demonstrated in other lepidopteran species where larvae can distinguish siblings from non-siblings through direct recognition mechanisms [22], (3) comparative silk properties between communal and solitary cocoons, (4) reciprocal transplant experiments between cinnamon and hog plum hosts, (5) comprehensive economic analysis of silk harvesting as pest management strategy, (6) phylogenetic analysis of gregarious cocooning evolution across Saturniidae, and (7) quantitative assessment of emergence success rates relative to position within cocoon clusters. Additional research should examine defensive efficacy of communal versus solitary cocoons against natural enemies, standardize semi-natural rearing protocols for cultivation, and conduct comparative studies with other gregarious pupating Saturniidae to understand evolutionary pathways of communal pupation strategies.

## 5. Conclusions

This study provides the first comprehensive documentation of the complete life cycle of *C. trifenestrata* Helfer on cinnamon (*Cinnamomum* spp.) as a novel host plant in Thailand. Laboratory conditions extended total life cycle duration (62.30 ± 3.68 days) compared to field conditions (56.30 ± 1.83 days), primarily through delayed egg and pupal stages. Despite a 40% larval size reduction on cinnamon, successful development and normal ontogenetic progression suggest a high degree of host adaptation plasticity.

Our controlled experiments provide evidence consistent with active gregarious behavior throughout development, with first-instar larvae frequently re-aggregating within 2–4 h (100% success rate, *n* = 5 trials) when experimentally scattered. The documentation of gregarious cocooning behavior, where 85.1% of individuals aggregated to construct individual cocoons within communal clusters, indicates a potential contribution to understanding social evolution in holometabolous insects. Pre-pupal larvae were observed traveling distances exceeding 50 cm to join aggregations, suggesting deliberate social tendencies rather than solely passive environmental channeling.

Environmental conditions influenced silk quality, with laboratory and hog plum cohorts producing higher cocoon shell ratios (5.02–5.30%) compared to field conditions (3.92%). The substantial yield from outbreak populations (>2 kg fresh weight) and efficient harvesting of communal clusters (individual clusters reaching 0.220 kg) indicate clear commercial potential. These findings support the possible development of community-based sericulture programs that transform pest species into sustainable bioresources, benefiting both rural communities and biodiversity conservation in Southeast Asia.

## Figures and Tables

**Table 1:** Developmental duration (mean ± SD in days) of *C. trifenestrata* Helfer life stages under three rearing conditions.

Developmental Stage	Field	Semi-Natural	Laboratory	F-Statistic	*p*-Value
Egg	9.50 ± 0.53 ^a^	9.40 ± 0.52 ^a^	11.00 ± 0.67 ^b^	24.371	<0.001
1st-Instar Larva	3.30 ± 0.48	3.60 ± 0.52	3.60 ± 0.52	1.174	0.324
2nd-Instar Larva	4.50 ± 0.53	4.60 ± 0.52	4.60 ± 0.52	0.123	0.885
3rd-Instar Larva	5.80 ± 0.79	6.20 ± 0.63	6.50 ± 0.71	2.431	0.107
4th-Instar Larva	6.90 ± 0.88	6.90 ± 0.88	7.00 ± 0.82	0.045	0.956
5th-Instar Larva	9.50 ± 0.53	9.40 ± 0.52	9.70 ± 0.48	0.9	0.418
Pupa	11.10 ± 0.88 ^a^	11.20 ± 1.03 ^a^	13.70 ± 1.42 ^b^	16.934	<0.001
Adult	4.70 ± 0.48 ^ab^	4.60 ± 0.52 ^a^	5.20 ± 0.42 ^b^	4.574	0.019
Total Duration	56.30 ± 1.83 ^a^	56.90 ± 1.60 ^a^	62.30 ± 3.68 ^b^	16.838	<0.001

Note: The sample size for each condition was *n* = 10 randomly selected individuals. Means within a row followed by different superscript letters (a, b) are significantly different (Tukey’s HSD test for individual stages, Games–Howell test for total duration, α = 0.05).

**Table 2:** Key morphological characteristics for stage identification.

Life Stage	Approx. Length/Wingspan (cm)	Distinguishing Features
Egg	0.12 ± 0.02	Ivory-white; oval and dorsoventrally flattened, resembling beads on a garland; laid in 1–3 neat parallel rows on the leaf edge.
1st-Instar Larva	0.58 ± 0.12	Cream-yellow body, later turning yellowish-red with dark transverse bands; prominent glossy dark head and dark-margined prothoracic plate; body covered in sparse, fine setae arising from light-brown scoli.
2nd-Instar Larva	0.65 ± 0.10	Body color a combination of red, yellow, and black; glossy brown head; golden-brown legs; clothed in a tuft of long white and short black hairs arising from brown scoli bearing golden spines.
3rd-Instar Larva	2.5 ± 0.11	Reddish-yellow body with yellow-black stripes; deep-red head capsule and prothoracic shield; legs are brick red; dense growth of long white setae arising from scoli that are encircled by a yellow band.
4th-Instar Larva	5.5 ± 0.1	Black body with vivid red-orange transverse bands; red head and anal claspers; prominent, long white setae become more conspicuous; body dots turn yellow.
5th-Instar Larva	7.5 ± 0.10	Black body with crimson-red transverse bands and glowing yellow dots; crimson-red head; covered with long whitish hairs projecting from pink tubercles (scoli); the yellow dots are luminescent under normal light and exhibit fluorescence under UV light.
Pupa	4.5 ± 0.13	Dark-brown in color; obtect, with a hard cuticle; female distinguished by a fine longitudinal line on the ventral side of the 8th abdominal segment, which is absent in the male.
Adult Male	Wingspan 6.5 ± 0.2	♂: Quadripectinate antennae; light golden-brown scales; forewing with one transparent fenestra and one small dark spot.
Adult Female	Wingspan 7.0 ± 0.02	♀: Bipectinate antennae (less feathery than male); dark brown and orange scales; forewing with three transparent fenestrae.

**Table 3:** Comparison of oviposition patterns between field and laboratory conditions.

Environment	Pattern Type	Sample Size	Frequency (%)	Examples (Eggs)	Substrate Types
Field	Linear (1–3 rows)	5 batches	100	34, 135, 169, 180, 260	Host leaves only
Laboratory	Small clusters	7/15 clusters	46.7	1, 2, 2, 3, 5, 6, 7	Mixed substrates *
Laboratory	Medium clusters	6/15 clusters	40	11, 14, 16, 25, 26, 30	Mixed substrates *
Laboratory	Large clusters	2/15 clusters	13.3	60, 98	Mixed substrates *

* Mixed substrates include host leaves, aluminum trays, mesh netting, and container floors. Sample represents random selection from numerous clusters observed throughout laboratory setup.

**Table 4:** Frequency distribution of cocoon cluster sizes across environmental conditions.

Cluster Size	Field	Semi-Field	Laboratory	Total (*n*)	Frequency (%)
Solitary (1)	3	2	2	7	14.9
Small (2–10)	12	5	6	23	48.9
Medium (11–30)	7	0	2	9	19.1
Large (31–100+)	8	0	0	8	17
Total	30	7	10	47	100

**Table 5:** Comparison of *C. trifenestrata* Helfer cocoon traits (mean ± SD) from four different rearing conditions (*n* = 5 for each sex in each condition).

Cocoon Trait	Field	Semi-Natural	Lab	Hog Plum	F-Statistic (Welch’s)	*p*-Value
Female						
Fresh cocoon weight (g)	1.724 ± 0.151	1.618 ± 0.178	1.604 ± 0.167	1.564 ± 0.103	1.102	0.399
Weight of pupa (g)	1.637 ± 0.139	1.522 ± 0.151	1.525 ± 0.131	1.466 ± 0.099	1.462	0.29
Cocoon shell weight (g)	0.066 ± 0.009 ^ab^	0.058 ± 0.018 ^a^	0.081 ± 0.017 ^b^	0.083 ± 0.006 ^b^	5.23	0.027
Cocoon shell ratio (%)	3.92 ± 0.51 ^a^	3.56 ± 0.67 ^a^	5.02 ± 0.72 ^b^	5.30 ± 0.30 ^b^	13.17	0.002
Male						
Fresh cocoon weight (g)	0.963 ± 0.227	0.941 ± 0.305	1.028 ± 0.192	1.009 ± 0.081	0.136	0.935
Weight of pupa (g)	0.917 ± 0.225	0.883 ± 0.297	0.944 ± 0.185	0.922 ± 0.078	0.045	0.986
Cocoon shell weight (g)	0.045 ± 0.017	0.046 ± 0.024	0.070 ± 0.011	0.059 ± 0.019	2.828	0.102
Cocoon shell ratio (%)	4.59 ± 1.03	4.96 ± 2.59	6.82 ± 0.78	5.85 ± 1.97	4.495	0.038

Note: For female traits, means within the same row followed by different superscript letters (a, b) are significantly different (Tukey HSD, *p* < 0.05). Post hoc tests for the significant male trait were not performed due to the violation of the homogeneity of variance assumption.

## Data Availability

The original contributions presented in this study are included in the article/Supplementary Materials. Further inquiries can be directed to the corresponding author.

## Supplementary Materials

The following supporting information can be downloaded at: https://www.mdpi.com/article/10.3390/insects16090914/s1, Video S1: Developmental stages of gregarious feeding—early instars (1st, 3rd) aggregating on single leaves and late instar (5th) feeding together on same branches despite abundant alternative sites; Video S2: Pre-pupal larvae demonstrating trail following behavior to locate and select suitable cocooning sites (in laboratory conditions); Video S3: Mature larvae demonstrating gregarious cocooning behavior in laboratory conditions.
